# Effect of telephone-based health education intervention models on cervical cancer screening compliance: A protocol for systematic review and meta-analysis

**DOI:** 10.1097/MD.0000000000022130

**Published:** 2020-12-04

**Authors:** Yinchun Liu, Qiang Zhang, Yanli Chen, Chun Wang

**Affiliations:** a Department of Emergency, Army Medical Center of PLA, Chongqing, China; b Department of Oncology, Army Medical Center of PLA, Chongqing, China; c Department of Gynecology, The First Hospital Affiliated tо AMU, Chongqing, China; d Department of General Practice and Health Examination Center, Army Medical Center of PLA, Chongqing, China.

**Keywords:** telephone-based health education intervention, cervical cancer screening, compliance, protocol, systematic review, meta-analysis

## Abstract

**Background:**

Screening is an effective strategy for preventing and controlling the cervical cancer. Unfortunately, women are often less likely to adhere to cervical cancer screening procedures. Related research shows that the telephone-based health education model can improve the compliance of screening. At present, however, this practice is lack of persuasion. Therefore, this study makes a systematic meta-analysis on whether the telephone-based health education model can improve the compliance of screening by women.

**Methods:**

Retrievals will be made on PubMed, Web of Science, the Cochrane Library, EMBASE, and some clinical trial registration websites, and information on related randomized controlled trials (RCTs) will collected. After 2 researchers independently screen the literatures, they will extract the data and evaluate the bias risk contained in the collected studies, before meta-analysis is carried out with RevMan 5.3 software.

**Results:**

The available evidence will be systematically reviewed in terms of compliance with cervical cancer screening.

**Conclusion:**

The findings of this study will produce comprehensive evidence to identify whether the telephone-based health education model can improve women's compliance with cervical cancer screening.

## 1 Introduction

Cervical cancer is a common malignant tumor with women. According to a report by the International Agency for Research on Cancer in 2018, the incidence and mortality of cervical cancer ranked fourth in the world. In 2018, there were about 570,000 new cases of cervical cancer, leading to about 311,000 deaths.^[1,2]^ As the only cancer with definite etiology, cervical cancer is preventable and treatable at present. Early detection of it can be improved by raising awareness, conducting early counseling with health professionals and implementing screening programs.^[3]^

Cervical cancer screening was introduced in Australia in the 1960s as the National Cervical Cancer Screening Program, jointly implemented by national, state, and regional governments to provide free screening services for women of the right ages. Aimed at women between the ages of 18 and 69 years who have a sexual history, the program requires a screening interval of once every 2 years. From 1991 to 2002, the program has reduced the morbidity and mortality of cervical cancer by 50%, with both of them having since been stable.^[1]^

Despite extensive cancer screening being arranged, its compliance tends to be low.^[4,5]^ A clinical investigation has found that most women have not much knowledge on the disease, with poor awareness of early screening.^[6–8]^ Some investigations show that up to 23.5% of women have never been screened in their lifetime.^[9]^ Therefore, improving the awareness of the disease is conducive to improving the participation in cervical cancer screening.

At present, to improve the awareness and compliance of cervical cancer screening, different strategies have been implemented, including written letters, phone calls or text messages, small videos, pamphlets, mass media, and face-to-face education programs.^[10]^ But these interventions have their own advantages and disadvantages. How to reduce the cost and improve the compliance of cervical cancer screening is still a focus of the current research.

Studies have demonstrated that telephone-based health education can improve the compliance of people who do not insist on screening by 20%.^[11]^ However, given various forms of telephone-based interventions, results from different surveys are controversial. Therefore, this study will conduct a meta-analysis to explore the impact of telephone-based intervention model on women's cervical cancer screening compliance, so as to provide reliable evidence for the prevention of the disease.

## 2 Methods

### 2.1 Protocol register

This study's protocol is drafted under the guidance of the Preferred Reporting Items for Systematic Reviews and Meta-analyses Protocols (PRISMA-P).^[12]^ Moreover, it has been registered on OSF (Registration number: DOI 10.17605/OSF.IO/R8F45).

### 2.2 Ethics

As this is a protocol with no patient recruitment and personal information collection, the approval of the ethics committee is not required.

### 2.3 Eligibility criteria

#### 2.3.1 Types of studies

This study will collect all the available randomized controlled trials (RCTs) about telephone-based health education intervention models on cervical cancer screening compliance, regardless of blinding, publication status, region, and language.

#### 2.3.2 Research objects

Women aged between 18 and 65 years and eligible for cervical cancer screening are included in the study (who have initiated sexual activities, have not undergone hysterectomy, and have not received any treatment for cervical cancer).

#### 2.3.3 Intervention measures

The experimental group receives health education intervention by telephone, and the forms of health education are unlimited. Women in the control group are invited to participate in cervical cancer screening through a written letter that meets the standard nursing criteria.

#### 2.3.4 Outcome indicators

Compliance rate of screening.

### 2.4 Exclusion criteria

(1) Studies published repeatedly;
(2) Studies whose literature forms are abstracts or conference papers, with no ways to obtain the original data;
(3) Studies whose data are incomplete or have obvious errors that cannot be addressed after communications with the authors;
(4) Studies with wrong random methods.

### 2.5 Retrieval strategy

Such terms as “health education,” “telephone,” “cervical cancer,” and “screening” are searched in the following databases: PubMed, Web of Science, the Cochrane Library, EMBASE, and some clinical trial registration websites. The retrieval period is from the establishment of databases to July 2020, and all RCTs on telephone-based health education intervention models related to cervical cancer screening compliance are collected. Taking PubMed as an example, the retrieval strategy is summarized in Table 1.

**Table 1:** Search strategy in PubMed database.

Number	Search terms
#1	telephone intervention[Title/Abstract]
#2	telephone-based intervention[Title/Abstract]
#3	telephone interview[Title/Abstract]
#4	telephone consultation[Title/Abstract]
#5	mobile telephone[Title/Abstract]
#6	#1 OR #2 OR #3 OR #4 OR #5
#7	Uterine Cervical Neoplasms[Mesh]
#8	Cancer of Cervix[Title/Abstract]
#9	Cancer of the Cervix [Title/Abstract]
#10	Cancer of the Uterine Cervix[Title/Abstract]
#11	Cervical Cancer[Title/Abstract]
#12	Cervical Neoplasms[Title/Abstract]
#13	Cervix Cancer[Title/Abstract]
#14	Cervix Neoplasms[Title/Abstract]
#15	Neoplasms, Cervical[Title/Abstract]
#16	Neoplasms, Cervix[Title/Abstract]
#17	Uterine Cervical Cancer[Title/Abstract]
#18	Cancer, Cervix[Title/Abstract]
#19	Cancer, Uterine Cervical[Title/Abstract]
#20	Cancers, Cervix[Title/Abstract]
#21	Cancers, Uterine Cervical[Title/Abstract]
#22	Cervical Cancer, Uterine[Title/Abstract]
#23	Cervical Cancers, Uterine[Title/Abstract]
#24	Cervical Neoplasm[Title/Abstract]
#25	Cervical Neoplasm, Uterine[Title/Abstract]
#26	Cervical Neoplasms, Uterine[Title/Abstract]
#27	Cervix Neoplasm[Title/Abstract]
#28	Neoplasm, Cervical[Title/Abstract]
#29	Neoplasm, Cervix[Title/Abstract]
#30	Neoplasm, Uterine Cervical[Title/Abstract]
#31	Neoplasms, Uterine Cervical[Title/Abstract]
#32	Uterine Cervical Cancers[Title/Abstract]
#33	Uterine Cervical Neoplasm[Title/Abstract]
#34	#7 OR #8 OR #9 OR #10 OR #11 OR #12 OR #13 OR #14 OR #15 OR #16 OR #17 OR #18 OR #19 OR #20 OR #21 OR #22 OR #23 OR #24 OR #25 OR #26 OR #27 OR #28 OR #29 OR #30 OR #31OR #32 OR #33
#35	Patient Compliance[Mesh]
#36	Patient Adherence[Title/Abstract]
#37	Patient Cooperation[Title/Abstract]
#38	Patient Noncompliance[Title/Abstract]
#39	Patient Non-Adherence[Title/Abstract]
#40	Patient Non-Compliance[Title/Abstract]
#41	Patient Nonadherence[Title/Abstract]
#42	Adherence, Patient[Title/Abstract]
#43	Compliance, Patient[Title/Abstract]
#44	Cooperation, Patient[Title/Abstract]
#45	Non-Adherence, Patient[Title/Abstract]
#46	Non-Compliance, Patient[Title/Abstract]
#47	Nonadherence, Patient[Title/Abstract]
#48	Noncompliance, Patient[Title/Abstract]
#49	Patient Non Adherence[Title/Abstract]
#50	Patient Non Compliance[Title/Abstract]
#51	# 35 OR #36 OR #37 OR #38 OR #39 OR #40 OR #41 OR #42 OR #43 OR #44 OR #45 OR #46 OR #47 OR #48 OR #49 OR #50
#52	Mass Screening[Mesh]
#53	Screening[Title/Abstract]
#54	Mass Screenings[Title/Abstract]
#55	Screening, Mass[Title/Abstract]
#56	Screenings[Title/Abstract]
#57	Screenings, Mass[Title/Abstract]
#58	#52 OR #53 OR #54 OR #55 OR #56 OR #57 OR #58
#59	#6 AND #34 AND #51 AND #58

### 2.6 Data screening and extraction

**Figure 1:**
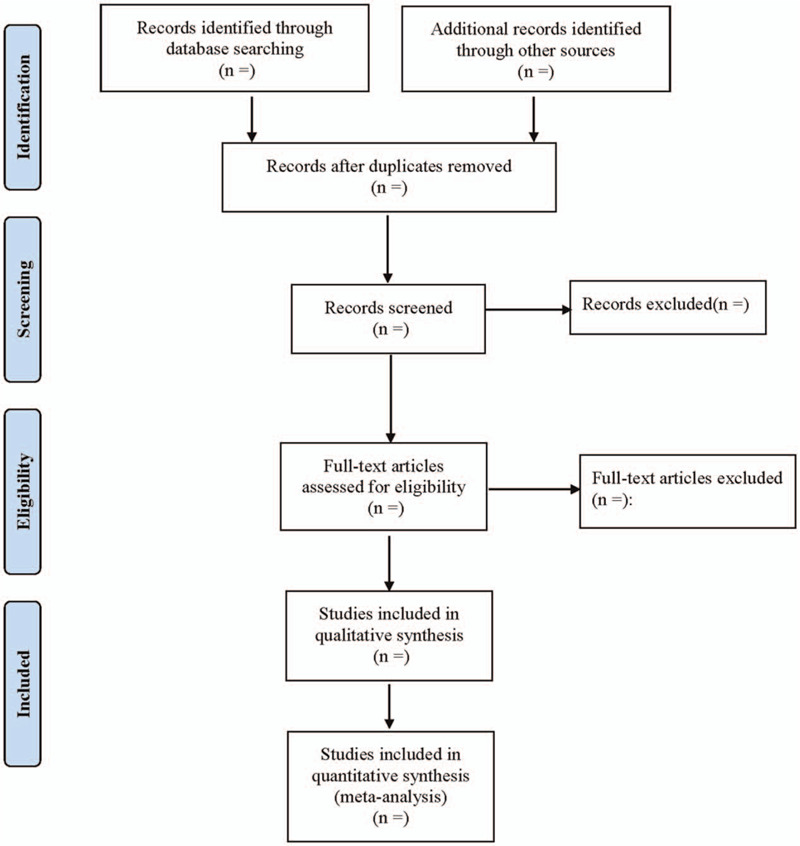
Flow diagram of study selection process.

According to the inclusion and exclusion criteria, 2 researchers will independently screen the literatures and cross-check the extracted data, and the blind method is used in the whole screening process. If any literature with disputes found in the screening is not sure whether to be included, it shall be addressed through the group discussion or decided by a third researcher. The extracted messages include the first author, publication time, sample size, outcome indicators, intervention measures, measurement tools, evaluation time of outcome indicators, main results, intervention personnel, etc. The literature screening process is shown in Figure 1.

### 2.7 Literature quality evaluation

Two researchers will independently evaluate the literature quality by using a bias risk assessment tool following the quality evaluation standard as specified in the Cochrane handbook. The risks are divided into 3 levels according to the probability of bias risks. Complete satisfaction means that the risk of bias is the smallest, rated as Grade A; partial satisfaction means that the possibility of bias is moderate, rated as Grade B; complete dissatisfaction means the possibility of bias is high, rated as Grade C.

### 2.8 Statistical analysis

#### 2.8.1 Data analysis and processing

The RevMan 5.3 software (CochraneCommunity, London, UK) provided by the Cochrane Collaboration is used for statistical analysis. Relative risk (RR) is used for statistics, and all the above data are represented by effect value at 95% confidence intervals (CIs). Heterogeneity test: Q test is used to qualitatively determine interstudy heterogeneity: If *P* ≥ .1, there is no interstudy heterogeneity; if *P* < .1, there is interstudy heterogeneity. At the same time, *I*^2^ value is used to quantitatively evaluate the interstudy heterogeneity: If *I*^2^ ≤ 50%, the heterogeneity is considered to be good, and the fixed-effect model will be adopted; if *I*^2^ > 50%, it shows significant heterogeneity, and the source of heterogeneity would be explored through subgroup analysis or sensitivity analysis. If there was no obvious clinical or methodological heterogeneity, it would be considered as statistical heterogeneity, and the random-effect model would be used for analysis. Descriptive analysis will be used if there was significant clinical heterogeneity between the 2 groups, and subgroup analysis is not required.

#### 2.8.2 Dealing with missing data

If data are missing or incomplete, the corresponding authors will be contacted to retrieve the missing data. If failed, such studies will be removed.

#### 2.8.3 Subgroup analysis

A subgroup analysis is conducted based on the age, education level, and intervention mode of the screened women.

#### 2.8.4 Sensitivity analysis

In order to test the stability of meta-analysis results of outcomes, a one-by-one elimination method will be adopted for sensitivity analysis.

#### 2.8.5 Assessment of reporting bias

Egger and Begg tests are used to quantitatively assess the potential publication bias.

#### 2.8.6 Evidence quality evaluation

The Grading of Recommendations Assessment, Development, and Evaluation (GRADE) will be used to assess the quality of evidence.

## 3 Discussion

In 2018, there were an estimated 570,000 new cases of cervical cancer on a global scale, most of them occurring with women who either had no access to cervical screening, or had not participated in screening in regions where the program is available.^[13]^ Evidence-based medical surveys show that screening is an effective means for the prevention and treatment of cervical cancer.^[14]^ Early diagnoses and standardized treatments of cervical cancer and its precancerous lesions can effectively reduce the incidence and mortality of cervical cancer.^[15,16]^

However, the clinical surveys have found that many female patients usually do not have undergone regular physical examination. As a result, in the clinical diagnosis, most of the treatments are too late, and the best time for treatment has long been missed, thus increasing the case fatality rate.^[17–19]^ Therefore, it is of great significance to prevent the deterioration of cervical cancer by early screening and strengthening health education for potential patients with cervical cancer.

At present, there are many reports about the impact of telephone-based health education intervention models on the compliance with cervical cancer screening by women, but there is a lack of systematic and correct evaluations. Consequently, it is necessary to use evidence-based medicine to objectively evaluate the impact of telephone-based health education intervention models on women's cervical cancer screening compliance, so as to promote screening practice and provide scientific and evidence-based intervention strategies for this disease's prevention. However, this study also has some limitations. For example, different phone-based health education models will have diversified impacts on the results. In addition, at different levels of education, women's understanding of the disease varies as well. In addition, due to the limitation in language abilities, researches in other languages may be ignored, thus possibly leading to some publication biases.

## Author contributions

**Data collection:** Yinchun Liu and Qiang Zhang

**Funding support:** Qiang Zhang

**Literature retrieval:** Yanli Chen

**Software operating:** Yanli Chen

**Supervision:** Yanli Chen

**Writing – original draft:** Chun Wang and Yanli Chen

**Writing – review & editing:** Chun Wang and Yinchun Liu
